# The use of endobronchial ultrasound-guided transbronchial needle aspiration in the diagnosis of thyroid lesions

**DOI:** 10.1186/1472-6823-14-88

**Published:** 2014-11-22

**Authors:** Roberto F Casal, Mimi N Phan, Keerthana Keshava, Jose M Garcia, Horiana Grosu, D Ray Lazarus, Juan Iribarren, Daniel G Rosen

**Affiliations:** Section of Pulmonary and Critical Care Medicine, Baylor College of Medicine, Michael E. DeBakey VA Medical Center, 2002 Holcombe Blvd. Pulmonary Section 111i, Houston, TX 77030 USA; Division of Pulmonary and Critical Care Medicine, New York Methodist Hospital, Brooklyn, NY USA; Division of Endocrinology, Diabetes and Metabolism, Baylor College of Medicine, Houston, TX USA; Department of Pathology and Immunology, Baylor College of Medicine, Houston, TX USA

**Keywords:** EBUS-TBNA, Thyroid, Intrathoracic goiter

## Abstract

**Background:**

Non-palpable thyroid nodules can be difficult to access by conventional ultrasound-guided fine needle aspiration, particularly when they are intrathoracic. Many of these patients are subject to multiple follow up scans or invasive diagnostic procedures such as mediastinoscopy or surgical resection. We aim to describe the feasibility of endobronchial ultrasound-guided transbronchial needle aspiration (EBUS-TBNA) for diagnosis of thyroid lesions.

**Methods:**

All EBUS-TBNA performed at our institutions from February 2010 to February 2013 were screened, and those in which a thyroid biopsy was performed were reviewed.

**Results:**

We identified 12 cases of EBUS-TBNA thyroid biopsy. Nine patients had an indication for EBUS in addition to their thyroid lesions. The median age was 64 years (range 44 to 84 years), and 10 patients were male. Median lesion size was 22.5 mm (range, 10 to 43 mm). Five lesions were strictly intrathoracic. All cases were sampled with a 22G needle and rapid on-site cytologic examination. Adequate samples were obtained in all 12 cases. Malignancy was identified in 3 of the 12 patients (metastatic breast adenocarcinoma, large B-cell lymphoma, and metastatic lung adenocarcinoma). The remaining 9 samples were deemed to be benign nodules. Seven of these were confirmed by clinical follow-up (n = 3), biopsies (n = 3), or surgery (n = 1).

There were no EBUS-related complications.

**Conclusions:**

EBUS-TBNA might be a safe and effective alternative for sampling thyroid lesions, particularly useful for those located below the thoracic inlet. Further prospective studies are required to compare its diagnostic yield and safety profile with standard techniques.

## Background

Thyroid nodules occur in about 5-7% of the population, and of those, about 5% turn out to be malignant. Malignancy can be diagnosed through ultrasound-guided fine needle aspiration (US- FNA) with a low rate of complications and adequate samples for diagnosis in about 80% of cases, making it the procedure of choice [1–3]. Nevertheless, access to lesions that lie near the thoracic inlet or within the thoracic cavity can be difficult, risky, or even impossible with US- FNA. These cases typically lead to multiple follow up images or more invasive diagnostic modalities such as mediastinoscopy or surgical excision.

Endobronchial ultrasound-guided transbronchial needle aspiration (EBUS-TBNA) is now a well- established technique for sampling peribronchial and paratracheal lymph nodes and masses [4, 5]. It plays a key role in mediastinal staging of lung cancer with comparable and even superior diagnostic yield than mediastinoscopy, and with an excellent safety profile [6–8]. Literature review reveals only a few case reports of EBUS-TBNA for the diagnosis of intrathoracic thyroid lesions [9–11]. The aim of this study is to describe our experience of EBUS-TBNA for the sampling of thyroid lesions in terms of feasibility, diagnostic yield and safety profile.

## Methods

After obtaining IRB approval, medical records from all EBUS-TBNA procedures performed on patients older than 18 years of age at the Michael E. DeBakey VA Medical Center (Houston, Texas) and at the New York Methodist Hospital (Brooklyn, New York) from February 2010 to February 2013 were reviewed. Due to the retrospective nature of the study, patient consent was waived by our local IRB (Institutional Review Board). We retrieved and analyzed those cases in which a thyroid biopsy was performed with EBUS-TBNA. Demographic and clinical data were obtained, including: baseline thyroid disease, size and location of thyroid lesion, indications for bronchoscopy, pre-procedure diagnoses, imaging reports prior to bronchoscopy and up to 12 months afterwards when available, description of techniques used in acquiring and processing the biopsy, documented complications of biopsy acquisition, pathology reports, and clinical reports up to 12 months after EBUS-TBNA. The result provided by EBUS-TBNA biopsy was compared to that of surgically removed thyroid specimens or US-FNA when available. Otherwise, the patient’s clinical course and follow up images for up to 12 months were examined to determine if there was a correlation with the original diagnosis.

## Results

**Figure 1:**
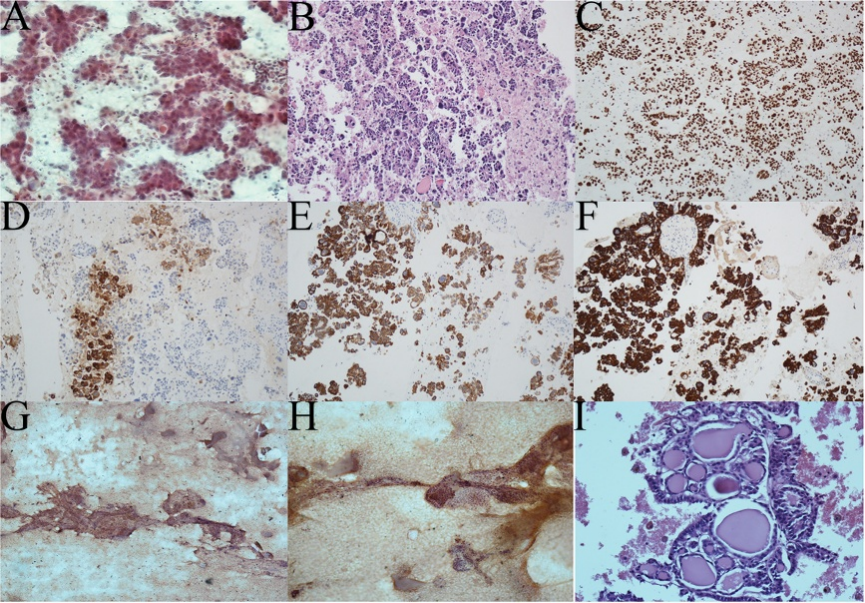
**Representative microphotographs from EBUS-guided thyroid aspirations.** Representative microphotographs from EBUS procedure: **(A)** papanicoulau stained smear and **(B)** cell block showing a poorly differentiated adenocarcinoma with papillary features. Immunohistochemistry showed these tumor cells with strong and diffuse positivity for TTF1 **(C)**, focal staining for carcinoembryonic antigen **(D)**, strong diffuse positivivty for CK7 **(E)**, and strong diffuse positivity for pancytokeratin. **(G)** and **(H)** show papanicoulau stained smear with clusters of benign follicular cells and colloid. **(I)** Cell block preparation showing thyroid follicles with colloid consistent with follicular nodule.

We identified 12 cases of EBUS-TBNA thyroid biopsy. In 9 patients, the primary indication for EBUS-TBNA was sampling of mediastinal lymph nodes or masses, and biopsy of the thyroid was done in addition to this during the same procedure. In the remainder 3, biopsy of an intrathoracic thyroid lesion was the only indication for EBUS-TBNA. Eleven patients underwent the procedure via laryngeal mask airway under general anesthesia and one under moderate sedation via oropharynx. In all cases biopsies were performed using a real-time ultrasound biopsy bronchoscope (XBF-UC260F-OL8; Olympus Ltd.; Tokyo, Japan) in standard fashion. Only in one procedure the operator reported difficulty introducing the needle through the tracheal wall due to the acute angle necessary to reach the lesion. Samples were obtained with a dedicated 22-gauge needle (NA-201SX; Olympus Ltd.; Tokyo, Japan), with an average of 4 passes. On-site cytology examination was available in all procedures. Needle aspirates were smeared onto slides and air-dried or fixed in 95% alcohol, collecting the remaining aspirate for cell-block preparation. Slides were processed immediately using Romanowsky technique (Diff- Quik®) and/or Pap- staining. The characteristics of each patient are summarized in Table 1. The median age was 64 years (range 44 to 84 years), and 10 patients (83%) were male. Median lesion size in the short axis was 22.5 mm (range, 10 to 43 mm). Eleven lesions (92%) were on the left thyroid lobe and one on the right. Five lesions (42%) were strictly intrathoracic. Adequate samples were obtained in all 12 cases (see examples in Figure 1). Malignancy was identified in 3 (25%) patients: metastatic adenocarcinoma of the breast, large B-cell lymphoma, and metastatic adenocarcinoma of lung origin. There was thyroid tissue in the background of these biopsies confirming thyroid sampling. These were *de novo* diagnosis of malignancy for each patient. The remaining 9 samples were deemed to be benign lesions (follicular nodules = 8, multinodular goiter = 1,). Seven of these 9 benign diagnoses were confirmed by clinical-radiographic follow-up (n = 3), CT-guided or US-FNA (n = 3), or surgery (n = 1). Based on the clinical and radiographical (i.e. radionuclide scanning, follow-up ultrasound) behavior of these lesions, they were all presumed to be benign. The diagnosis of multinodular goiter was made with histopathology from surgical resection. There were no EBUS-related complications. Of note, one of the patients who underwent a subsequent US- FNA developed a large hematoma as a complication of the procedure.

**Table 1:** **Patient characteristics**

Subject	Baseline thyroid disease	Indication for procedure	Size (mm)**	Strictly intrathoracic	Diagnosis
1	Multinodular goiter	Multiple lung masses, enlarged mediastinal LNs	19	No	Follicular nodule
2	None	Lung mass w/ enlarged hilar LNs	26	No	Large B-cell lymphoma
3	None	Lung mass	20	No	Follicular nodule
4*	None	Enlarged mediastinal LNs	20	Yes	Metastatic breast adenocarcinoma
5	None	Superior mediastinal mass	25	Yes	Follicular nodule
6	None	Lung mass	18	No	Follicular nodule
7	Subclinical hypothyroidism	Lung mass	33	Yes	Follicular nodule
8	Subclinical hyperthyroidism	Enlarged mediastinal LNs	32	Yes	Follicular nodule
9	None	Lung mass, enlarged mediastinal LNs	10	No	Metastatic lung adenocarcinoma
10	None	Lung mass w/ enlarged hilar LNs	33	No	Follicular nodule
11	None	Substernal thyroid mass	44	No	Follicular nodule
12*	None	Incidentally found anterior mediastinal mass	16	Yes	Multinodular goiter

*Patients number 4 and 12 were the only female in this study.

**Measured in the short axis.

## Discussion

Ultrasound-guided FNA is the procedure of choice for sampling of thyroid nodules [12]. Nevertheless, for those lesions located below the thoracic inlet, the procedure becomes challenging and it imposes a higher risk since the needle trajectory is close to many vital structures. Our case series demonstrates that EBUS-TBNA is a feasible diagnostic approach for these lesions, being potentially safe and effective as well.

As previously mentioned, US-FNA provides diagnostically useful information in about 80% of thyroid nodules and has an average sensitivity of about 95% in patients with malignant thyroid nodules [13]. In our limited experience, EBUS-TBNA has provided cytologic samples comparable to those of US-FNA. Moreover, the EBUS scope also allowed us to characterize the gland and lesion in question providing ultrasonographic characteristics that can predict malignancy: solid mass, microcalcifications, increased vascularity, irregular borders [3]. Nevertheless, the authors could not over emphasize the fact that the decision to sample any thyroid lesion should always be made together with the managing endocrinologist. The diagnostic work up of the thyroid nodule has been extensively reviewed elsewhere in the literature and it is not within the scope of our manuscript [1, 3, 13, 14].

Our malignancy rate was much higher (3/12, 25%) than that expected for thyroid nodules in the general population (5%). Moreover, all malignancies were metastatic to the thyroid, and none were primary thyroid neoplasms. We, indeed, found the most common non-thyroid malignancies that involve the thyroid: lymphoma, breast cancer, and lung cancer. This is likely because our population was referred for EBUS-TBNA of mediastinal lymph nodes or masses, hence with a much higher pre-test probability of having a malignancy outside the thyroid. The need to sample the thyroid, in addition to their lung or mediastinal lesions, was to rule out a second primary cancer. Four of our patients had an incidental finding of an 18-fluorodeoxyglucose avid thyroid nodule by positron emission tomography (PET) scan. Three of these four were confirmed to be malignant with EBUS-TBNA. A systematic review from Shie and coworkers found the prevalence of incidental focal abnormalities detected by PET in the thyroid gland to be roughly 1% (571 from 55160 patients) [15]. Of these incidentally found thyroid nodules 33.2% were malignant in origin, which suggests that these findings need to be confirmed by cytology or histology.

Wakeley and Mulvany divided intrathoracic goiters into three types based on location and extent: “a”, small substernal extension of a mainly cervical thyroid goiter; “b”, partial intrathoracic goiter wherein the major portion of the goiter is situated within the thorax; and “c”, complete intrathoracic goiter wherein the goiter lies entirely within the thoracic cavity [16, 17]. Following this classification, in our small series, 5 patients were type “a”, 2 patients were type “b”, and 5 type “c”.

Ultrasound guided-FNA complications are typically minor and self-limited such as local pain and small hematomas. More severe complications occur in less than 5% of cases: large hematoma, infection, needle track seeding of malignancy, recurrent laryngeal nerve palsy [2]. EBUS-TBNA has also been demonstrated to be an extremely safe procedure with an overall complication rate of less than 2% [8]. Data on patients undergoing EBUS-TBNA in the American College of Chest Physicians Quality Improvement Registry, Evaluation and Education (AQuIRE) database found that out of 1,317 patients at six hospitals, complications occurred in 19 patients only. There were no infectious complications, and pneumothorax, which occurred in 7 patients, was in fact associated with the addition of transbronchial lung biopsy [8]. Of note, there has been a recent report of an EBUS-TBNA biopsy of a cystic thyroid nodule which resulted in a thyroid abscess [18]. Unlike US-FNA, a completely aseptic technique, EBUS- TBNA implies passing the needle through the working channel of the bronchoscope that might be contaminated with oropharyngeal flora and/or tracheobronchial secretions. Physicians should be aware of this potential risk of infection, and cystic lesions in particular should be avoided.

## Conclusions

To the best of our knowledge, this is the first series to describe the use of EBUS-TBNA for the sampling of thyroid lesions. In patients with intrathoracic thyroid lesions, EBUS-TBNA could potentially prevent complications associated with more invasive procedures, or reduce cost and anxiety associated with close follow-up and repeat imaging. We hope our results will prompt prospective comparisons between EBUS-TBNA, US-FNA and surgical approach to thoroughly assess its yield and safety profile. Until then, EBUS-TBNA should be reserved for intrathoracic thyroid lesions that are not amenable for US-FNA.

## Abbreviations

EBUS-TBNA: Endobronchial ultrasound-guided transbronchial needle aspiration
US-FNA: Ultrasound-guided fine needle aspiration
PET: Positron emission tomography.

## References

[1] Hegedus L (2004). The thyroid nodule. N Engl J Med.

[2] Polyzos SA, Anastasilakis AD (2009). Clinical complications following thyroid fine-needle biopsy: a systematic review. Clin Endocrinol (Oxf).

[3] Cooper DS, Doherty GM, Haugen BR, Kloos RT, Lee SL, Mandel SJ, Mazzaferri EL, McIver B, Pacini F, Schlumberger M, Sherman SI, Steward DL, Tuttle RM (2009). Revised American Thyroid Association management guidelines for patients with thyroid nodules and differentiated thyroid cancer. Thyroid.

[4] Herth FJ, Eberhardt R, Vilmann P, Krasnik M, Ernst A (2006). Real-time endobronchial ultrasound guided transbronchial needle aspiration for sampling mediastinal lymph nodes. Thorax.

[5] Yasufuku K, Chiyo M, Sekine Y, Chhajed PN, Shibuya K, Iizasa T, Fujisawa T (2004). Real- time endobronchial ultrasound-guided transbronchial needle aspiration of mediastinal and hilar lymph nodes. Chest.

[6] Ernst AAD, Eberhardt R, Krasnik M, Herth FJ (2008). Diagnosis of mediastinal adenopathy- real-time endobronchial ultrasound guided needle aspiration versus mediastinoscopy. J Thorac Oncol.

[7] Yasufuku K, Pierre A, Darling G, de Perrot M, Waddell T, Johnston M, da Cunha Santos G, Geddie W, Boerner S, Le LW, Keshavjee S (2011). A prospective controlled trial of endobronchial ultrasound-guided transbronchial needle aspiration compared with mediastinoscopy for mediastinal lymph node staging of lung cancer. J Thorac Cardiovasc Surg.

[8] Eapen GA, Shah AM, Lei X, Jimenez CA, Morice RC, Yarmus L, Filner J, Ray C, Michaud G, Greenhill SR, Sarkiss M, Casal R, Rice D (2013). Complications, consequences, and practice patterns of endobronchial ultrasound-guided transbronchial needle aspiration: results of the AQuIRE registry. Chest.

[9] Chalhoub MHK (2010). The use of endobronchial ultrasonography with transbronchial needle aspiration to sample a solitary substernal thyroid nodule. Chest.

[10] Jeebun VNS, Harrison R (2009). Diagnosis of a posterior mediastinal goitre via endobronchial ultrasound-guided transbronchial needle aspiration. Eur Respir J.

[11] Chalhoub M, Harris K (2012). Endobronchial ultrasonography with transbronchial needle aspiration to sample a solitary substernal thyroid nodule: a new approach. Heart Lung Circ.

[12] La Rosa GL, Belfiore A, Giuffrida D, Sicurella C, Ippolito O, Russo G, Vigneri R (1991). Evaluation of the fine needle aspiration biopsy in the preoperative selection of cold thyroid nodules. Cancer.

[13] Burch HB (1995). Evaluation and management of the solid thyroid nodule. Endocrinol Metab Clin North Am.

[14] Tan GH, Gharib H (1997). Thyroid incidentalomas: management approaches to nonpalpable nodules discovered incidentally on thyroid imaging. Ann Intern Med.

[15] Shie P, Cardarelli R, Sprawls K, Fulda KG, Taur A (2009). Systematic review: prevalence of malignant incidental thyroid nodules identified on fluorine18 fluorodeoxyglucose positron emission tomography. Nucl Med Commun.

[16] Wakely CPG, Mulvaney JH (1940). Intrathoracic goiter. SGO.

[17] Shields TW (2005). General Thoracic Surgery. Sixth Edition. Chapter 168.

[18] Kennedy MP, Breen M, O’Regan K, McCarthy J, Horgan M, Henry MT (2012). Endobronchial ultrasound-guided transbronchial needle aspiration of thyroid nodules: pushing the boundary too far?. Chest.

[CR19] The pre-publication history for this paper can be accessed here:http://www.biomedcentral.com/1472-6823/14/88/prepub

